# Intra- and Inter-Session Reliability of Average- and Trial-Level Motor Evoked Potential Amplitudes

**DOI:** 10.3390/brainsci16090911

**Published:** 2026-08-27

**Authors:** Renming Liu, Aung Aung Kywe Moe, Zhen Zheng, Shapour Jaberzadeh

**Affiliations:** 1 Monash Neuromodulation Research Unit, Department of Physiotherapy, School of Primary and Allied Health Care, Peninsula Campus, Monash University, Melbourne, VIC 3199, Australia; 2 Department of Medical Imaging and Radiation Sciences, Monash University, Melbourne, VIC 3199, Australia; 3 School of Health and Biomedical Sciences, STEM College, RMIT University, Melbourne, VIC 3083, Australia

**Keywords:** transcranial magnetic stimulation, motor evoked potential, corticospinal excitability, reliability study

## Abstract

**Highlights:**

**What are the main findings?**

- Averaging 25 motor evoked potentials (MEPs) produced excellent intra-session and good inter-session reliability for estimating corticospinal excitability in healthy adults.
- Individual MEP responses showed substantial trial-to-trial variability and wider limits of agreement, while exploratory analyses suggested variation across the recording sequence.

**What are the implications of the main findings?**

- Averaged MEP amplitude remains an appropriate primary outcome when the aim is to obtain a stable estimate of corticospinal excitability.
- Trial-level hierarchical modelling can complement averaged analyses by characterising response variability and trial-order effects that are not represented by the overall mean.

**Abstract:**

Background: Motor evoked potential (MEP) amplitude elicited by single-pulse transcranial magnetic stimulation (TMS) is widely used as an indirect index of corticospinal excitability (CSE). Averaged MEP amplitudes are commonly used to improve measurement stability, but averaging may obscure some trial-level information across the recording sequence. As trial-level and hierarchical modelling approaches become increasingly common, it remains important to determine how averaged and trial-level MEP amplitudes should be interpreted as measures of CSE. Objective: This study compared the intra-session and inter-session reliability of averaged and trial-level MEP amplitudes, with exploratory block analyses used to examine whether trial-order structure was obscured by averaging. Methods: Twelve healthy adults completed two sessions with three CSE assessments at T1, T2, and T3 (T1 and T2 20 min apart; T3 at least 48 h later). At each time point, 25 single-pulse MEPs were elicited at 120% resting motor threshold and recorded from the right first dorsal interosseous muscle. Reliability was assessed using intraclass correlation coefficients (ICC), coefficients of variation (CV), minimal detectable change at the 95% confidence level (MDC95), Bland–Altman analysis, linear mixed-effects models, exploratory sequential block analyses, and Bayesian variance decomposition. Results: Average MEP amplitudes remained stable across T1, T2, and T3. Average-level reliability was good across all timepoints (ICC = 0.899) and excellent within session (T1 vs. T2: ICC = 0.992, CV = 4.935%, MDC95 = 0.191 mV). Inter-session average-level reliability remained good (ICC = 0.844–0.852). In contrast, trial-level analyses revealed substantial within-participant variability, with low ICCs, high CVs, and large MDC95 values. Bland–Altman analyses showed minimal mean bias but substantially wider limits of agreement for trial-level data. Exploratory sequential block analyses showed that, in the late block, T1 differs significantly from T3. Bayesian modelling indicated that residual trial-level variability exceeded between-subject variability, with a posterior ICC of 0.336. Conclusions: Averaging 25 MEPs provides a reliable estimate of corticospinal excitability, but it masks variability. Trial-level and exploratory block analyses suggest that this variability may contain meaningful temporal structure, supporting trial-level modelling as a complementary approach for validating and interpreting averaged MEP estimates.

## 1. Introduction

Corticospinal excitability (CSE) is a central neurophysiological marker for understanding the functional state of the human motor system [1]. It reflects the responsiveness of the corticospinal pathway to external stimulation and is widely used to investigate motor control, motor learning, neuroplasticity, and recovery after neurological injury [2]. In clinical and rehabilitation research, CSE provides a non-invasive insight into how the motor system changes in response to disease, training, pain, fatigue, pharmacological modulation, and therapeutic interventions [3,4,5]. Because many clinical studies aim to modify neural excitability or promote adaptive plasticity, reliable measurement of CSE is essential for determining whether an observed change represents a true physiological effect or merely measurement noise.

Transcranial magnetic stimulation (TMS) is one of the most commonly used methods for quantifying CSE [6]. When single-pulse TMS is applied over the primary motor cortex, it generates motor evoked potentials (MEPs) that can be recorded from target muscles using surface electromyography (EMG), with peak-to-peak amplitude serving as a common indirect measure of CSE [7].

However, MEP amplitude is inherently variable. A single MEP does not reflect cortical excitability alone, but rather the combined influence of cortical, spinal, peripheral, technical, and state-dependent factors [8]. Variability may arise from fluctuations in cortical neuronal excitability, spinal motor neuron responsiveness, background muscle activity, attention, arousal, coil position, stimulation intensity, and electrode placement [9]. As a result, MEP amplitudes can vary substantially from trial to trial even under carefully controlled experimental conditions. This variability creates a major methodological challenge: if MEP amplitudes are to guide clinical interpretation or intervention evaluation, their measurement must be sufficiently reliable at the level of analysis, whether based on averaged responses or individual trials.

Most studies have addressed MEP variability by averaging multiple trials within a block and using the mean amplitude as the unit of analysis [10,11]. This approach reduces trial-to-trial fluctuations and provides a more stable estimate of CSE. Previous work has therefore provided substantial evidence regarding the intra-session and inter-session reliability of mean MEP amplitude, including the influence of stimulation intensity, number of trials, and experimental standardization [12,13]. These studies have helped establish that averaged MEP amplitudes can provide acceptable to excellent reliability when protocols are carefully controlled. However, this approach also assumes that trial-to-trial variability can be treated primarily as nuisance noise and that the mean adequately represents the underlying response pattern.

Nevertheless, the field is increasingly moving beyond mean-based summaries. Averaging collapses the distribution of MEP responses into a single value and may obscure skewness, extreme responses, non-stationarity across the recording sequence, and changes in within-participant variability [14]. These features may be clinically and physiologically relevant, particularly when the research question concerns response variability, individual adaptation, or dynamic changes in corticospinal output [8]. Approaches such as linear mixed-effects models (LMMs) and Bayesian hierarchical models allow trial-level data to be retained while accounting for the nesting of repeated trials within participants [15,16,17]. This shift raises a key unresolved question: averaged MEP amplitudes may be reliable as summary outcomes, but do they adequately represent the structure of the underlying trial-level data? Directly comparing average-level and trial-level reliability is therefore necessary to clarify how MEP data should be analyzed and interpreted in clinical and experimental settings, while exploratory block analyses can help determine whether trial-order structure is hidden by full-block averaging.

Furthermore, the number of MEPs collected per block critically influences reliability. Increasing the trial number improves the stability of CSE estimates, with 20 MEP amplitudes commonly recommended as a minimum for excellent reliability [18]. Evidence suggests that increasing the trial number is more effective than discarding early responses [19]. Accordingly, this study recorded 25 MEPs per timepoint, enabling direct comparison of reliability at both the average and trial levels.

In addition to comparing full-block averages with individual trial-level responses, it is important to examine whether reliability is consistent across different parts of the recording block. MEP amplitudes may fluctuate across a sequence of repeated stimuli because of changes in attention, arousal, coil stability, habituation, or other state-dependent factors [20]. If early, middle, and late portions of a recording show different levels of stability, a full 25-trial average may obscure meaningful within-block temporal variation. Sequential block analyses can therefore test whether the average is a representative summary of the whole recording or whether it masks time-dependent features of the MEP response.

Establishing reliability at both average and trial levels has important clinical implications because intervention efficacy, patient monitoring, and individualized treatment responses may be interpreted differently depending on the analytical framework employed. Therefore, the primary aim of this study was to compare the reliability of averaged and trial-level MEP amplitudes across repeated assessments of CSE. Intra-session (20 min) and inter-session (48 h) reliability were evaluated using complementary metrics. A secondary exploratory aim was to examine whether early, middle, and late portions of the 25-trial recording block revealed trial-order or temporal structure that may be obscured by full-block averaging.

## 2. Methods

### 2.1. Participants

This study included healthy, right-hand-dominant adults aged 18 to 50 who were eligible for TMS safety assessment and had no prior exposure to TMS. Exclusion criteria included neurological or psychiatric conditions; psychiatric medication use; incompatible implants, aneurysm clips, and pacemakers. To control for confounding factors, participants abstained from caffeine for two hours and alcohol or grapefruit juice for 24 h before any session [11].

This study was approved by the Human Research Ethics Committee of Monash University (project ID: 41104). All participants provided written informed consent before the experimental sessions and received reimbursements for their time.

### 2.2. Study Setup

**Figure 1:**
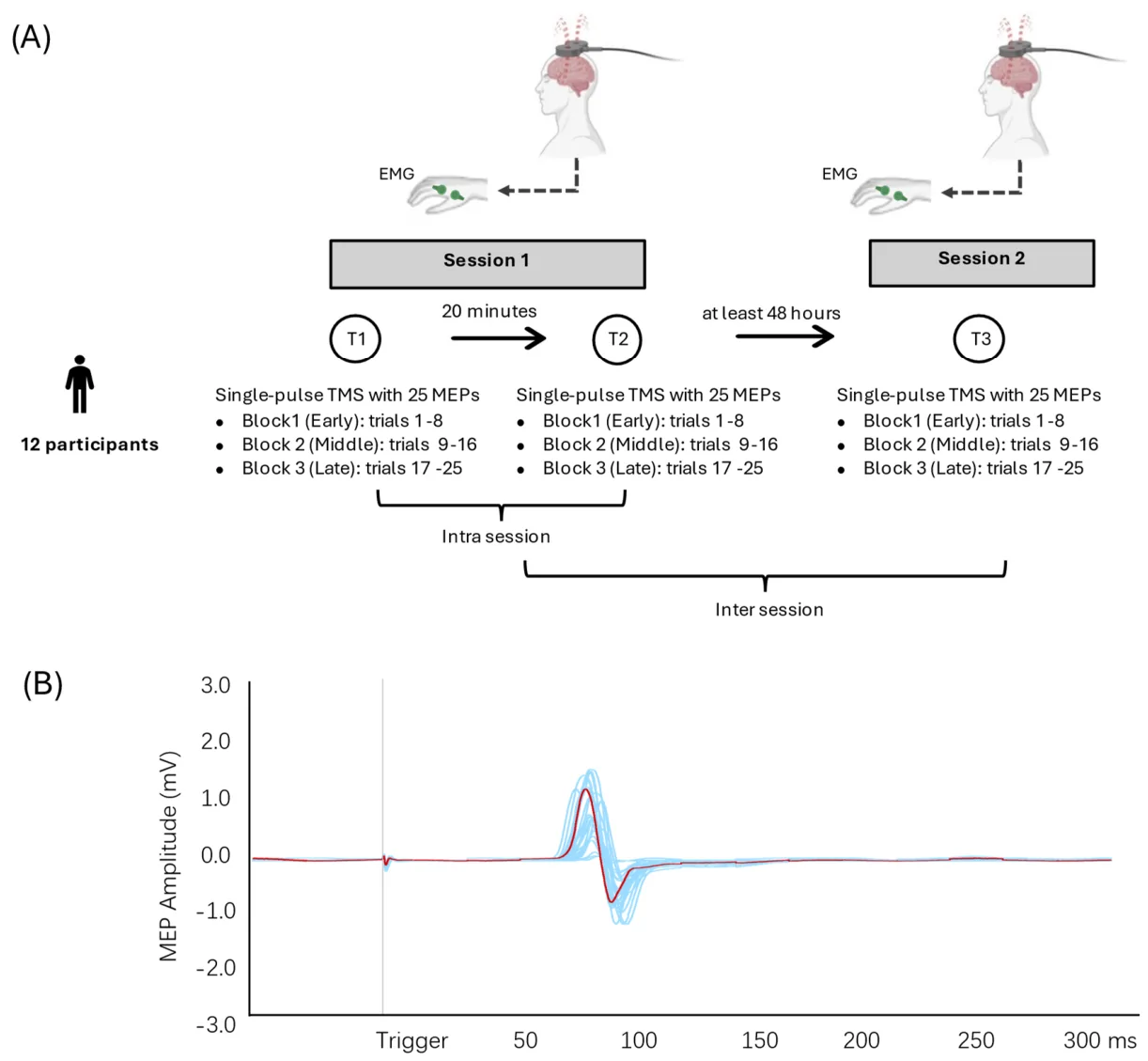
Experimental setup. (**A**) Single-pulse transcranial magnetic stimulation (TMS) was delivered over the primary motor cortex, and motor evoked potentials (MEPs) were recorded from the target hand muscle using electromyography (EMG). Each participant completed two sessions. In Session 1, 25 MEP trials were recorded at T1 and T2 to assess intra-session reliability. In Session 2, 25 MEP trials were recorded at T3 to assess inter-session reliability. The 25 MEP trials were further divided into three sequential blocks for exploratory analysis: Early, trials 1–8; Middle, trials 9–16; and Late, trials 17–25. T1 = time point 1; T2 = time point 2; T3 = time point 3. (**B**) Representative set of MEP waveforms from one participant at one assessment. Blue traces represent the 25 individual MEP trials, and the red trace represents the average waveform across these trials. The vertical line marks the TMS trigger. MEP amplitude was calculated as the peak-to-peak difference between the largest positive and negative EMG deflections after stimulation.

A repeated-measures within-subject design was used to examine the stability of CSE, indexed by peak-to-peak MEP amplitude. Each participant completed three MEP assessments labelled T1, T2, and T3, across two evaluation sessions, with all assessments performed by the same assessor. T1 and T2 were completed within the first experimental session and were separated by a 20-min interval to assess intra-session reliability. T3 was completed during a second experimental session conducted at least 48 h later to assess inter-session reliability [21]. To minimize the potential influence of circadian variation on CSE, the two experimental sessions were scheduled at the same time of day for each participant. During each assessment, participants were seated comfortably in an adjustable chair with both arms supported and the right hand relaxed. Participants were instructed to remain at rest throughout stimulation to minimize activities that could influence CSE (Figure 1).

### 2.3. MEP Recording and Processing

Surface EMG was recorded from the right first dorsal interosseous (FDI) muscle using disposable Ag/AgCl electrodes in a belly–tendon montage. Signals were amplified (×1000), band-pass filtered (10–500 Hz), digitized at 1 kHz using a PowerLab data-acquisition system (ADInstruments, Bella Vista, NSW, Australia), and recorded using LabChart software (version 8.1.31; ADInstruments, Bella Vista, NSW, Australia). The active electrode was placed over the muscle belly of the FDI, the reference electrode was placed over the proximal phalanx of the index finger, and a ground electrode was placed over a nearby bony prominence. Skin was prepared before electrode placement to reduce impedance, which was maintained below 5 kΩ. Participants were instructed to keep the target hand relaxed throughout testing, and background EMG activity was monitored visually and quantitatively prior to each stimulus; trials containing pre-stimulus muscle activation exceeding 0.01 mV were excluded from analysis before each stimulus. To ensure consistency across T1, T2, and T3, EMG electrodes were positioned by the same investigator using the same belly–tendon montage and anatomical landmarks of the right FDI muscle at each assessment.

The cortical representation of the FDI was identified over the left primary motor cortex (M1) using single-pulse TMS delivered via a MagPro R30 stimulator (MagVenture, Farum, Denmark) with a figure-eight coil. The coil was held tangentially to the scalp, and the stimulation hotspot was defined as the scalp location that elicited the largest and most consistent MEP amplitudes in the right FDI [22]. Resting motor threshold (RMT) was determined at the identified hotspot and defined as the lowest stimulation intensity required to evoke MEP amplitudes of at least 0.05 mV in at least 50% of trials. RMT was estimated using parameter estimation by sequential testing (PEST) with the TMS Motor Threshold Assessment Tool (MTAT 2.0; http://clinicalresearcher.org/software.htm) accessed on 26 December 2024. The same stimulation hotspot and coil orientation were used for repeated assessments within each participant, and stimulation intensity was kept constant across T1, T2, and T3.

At each assessment time point, 25 single-pulse TMS stimuli were delivered at 120% of RMT, with an interstimulus interval of 6 s, which could be used to prevent neural fatigue across trials. [23]. Stimulation intensity was kept constant across repeated assessments within each participant. MEP was calculated for each trial as the peak-to-peak difference between the maximum and minimum EMG deflection following stimulation. For average-level analyses, the 25 MEPs at each time point were averaged to generate one mean MEP amplitude per participant per assessment, representing the conventional summary estimate of CSE. For trial-level analyses, individual MEP amplitudes were retained as repeated observations to examine single-response reliability, within-participant variability, and information that may be lost when responses are averaged.

Exploratory sequential block analyses were conducted to support the interpretation of trial-level variability by examining whether response stability differed across the recording sequence. Each 25-trial recording was divided into three sequential blocks representing early, middle, and late responses: Block 1, trials 1–8; Block 2, trials 9–16; and Block 3, trials 17–25. For block-level average analyses, mean MEP was calculated separately within each block for each participant and time point. For block-level trial analyses, individual MEPs were retained within each block. These analyses were used to examine within-block temporal stability and to evaluate whether the full 25-trial average masked block-dependent variability, rather than to identify an optimal reduced number of trials.

### 2.4. Statistical Analysis

Statistical analyses and data visualization were conducted using GraphPad Prism (version 11.0.1) and R (version 4.5.2; R Foundation for Statistical Computing, Vienna, Austria). Descriptive statistics are presented as mean ± SD. LMMs were used to examine differences in MEP amplitudes across sessions (T1, T2, and T3), trials, and recording blocks while preserving the nested structure of the data. Participants were included as random intercepts to account for repeated observations within individuals, with session and trial order specified as fixed effects. This approach avoided treating repeated trials from the same participant as independent observations and allowed trial-level variability to be modelled directly. Model residuals were visually inspected to assess normality and homoscedasticity. Estimated marginal means, standard errors, t values, and *p* values were reported.

Reliability was assessed for both average-level and trial-level data using the intraclass correlation coefficient (ICC), coefficients of variation (CV), and the minimal detectable change at the 95% confidence level (MDC95). Average-level reliability was calculated from the mean MEP amplitude across trials within each session, whereas trial-level reliability was calculated from individual MEP amplitudes. ICC were calculated using a two-way mixed-effects, absolute-agreement model. ICC were interpreted according to established thresholds: poor (<0.50), moderate (0.50 to 0.75), good (0.75 to 0.90), and excellent (>0.90) [24]. Single-measure ICC were used for trial-level reliability, and average-measure ICC were used for average-level reliability. For the trial-level analysis, a Bayesian hierarchical model was applied to the trial-level data to estimate posterior ICC and decompose variance into between-subject and residual trial-level components.

Block-specific LMM and reliability analyses followed the same approach as the primary analyses. Timepoint comparisons were conducted separately for each sequential block, and ICC, CV, and MDC95 values were calculated for both block-averaged amplitudes and block-specific trial-level amplitudes. This allowed the stability of early, middle, and late responses within each recording block to be compared with the reliability of the full 25-trial average.

Agreement between timepoints was further evaluated using Bland–Altman analysis. For each comparison (T1 vs. T2, T1 vs. T3, and T2 vs. T3), the differences between paired measurements were plotted against their averages. Mean bias was calculated as the average difference between paired measurements, and the 95% limits of agreement were defined as mean bias ± 1.96 × the standard deviation of the differences, indicating the range within which most measurement differences were expected to fall.

Exploratory analyses examined associations between participant characteristics and MEP amplitudes. Age, sex, and BMI were entered as predictors. These analyses were considered exploratory.

## 3. Results

**Figure 2:**
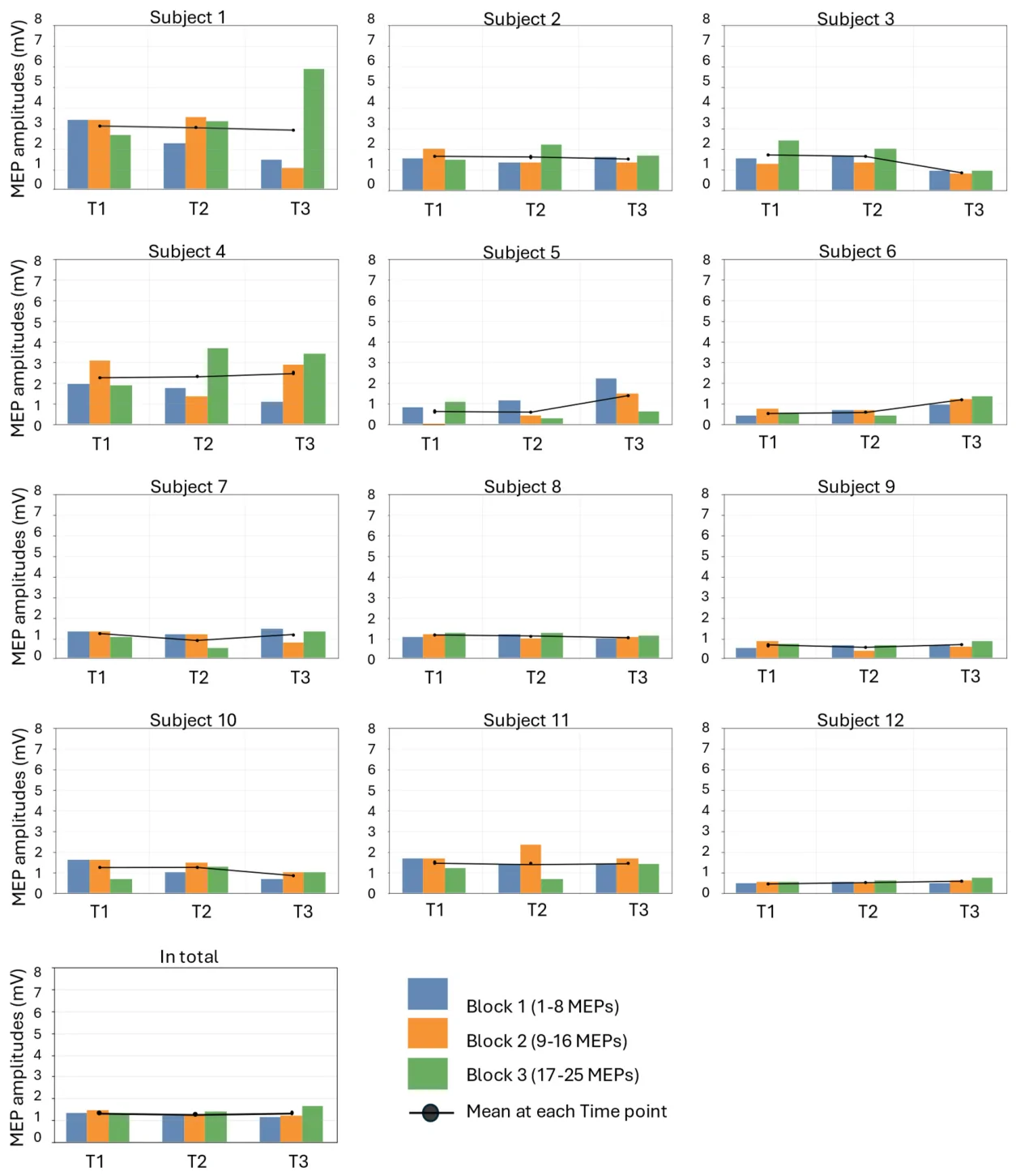
Individual and total mean MEP amplitudes across repeated assessments. Bars represent the mean peak-to-peak motor evoked potential (MEP) amplitudes for each participant at three assessment timepoints (T1, T2, and T3). T1 and T2 were conducted within the same session and separated by approximately 20 min to assess intra-session reliability, whereas T3 was conducted 48 h later to assess inter-session reliability.

Twelve participants (6 female [50%]; mean [SD] age, 29.42 [8.24] years) participated in this study. Across the 12 participants, mean peak-to-peak MEP amplitudes remained relatively stable across repeated assessments: T1 (1.35 ± 0.76 mV), T2 (1.31 ± 0.75 mV), and T3 (1.29 ± 0.67 mV). Detailed results for each participant are presented in Figure 2. RMT did not differ significantly between Session 1 and Session 2 (36.50 ± 5.23 vs. 37.83 ± 5.57; mean difference = 1.33, 95% CI: −0.63 to 3.29; t (11) = 1.50, *p* = 0.162) (Supplementary Material).

### 3.1. Average-Level Reliability

Average-level LMM analyses showed no significant differences in MEP amplitudes between timepoints (Table 1). Pairwise comparisons showed no significant difference for T1 vs. T2 (β = 0.045 mV, 95% CI: −0.208 to 0.298, t (22) = 0.450, *p* = 0.895), T1 vs. T3 (β = −0.003 mV, 95% CI: −0.255 to 0.250, t (22) = −0.025, *p* = 1.000), or T2 vs. T3 (β = −0.048 mV, 95% CI: −0.301 to 0.205, t (22) = −0.474, *p* = 0.884).

Overall reliability across the three timepoints was good, with an ICC of 0.899, a CV of 17.735%, and an MDC95 of 0.642 mV. Intra-session reliability between T1 and T2 was excellent, with an ICC of 0.992, a CV of 4.935%, and an MDC95 of 0.191 mV. Inter-session reliability was lower but remained good, with ICCs of 0.844 for T1 vs. T3 and 0.852 for T2 vs. T3. Corresponding CVs were 22.393% and 22.004%, and MDC95 values were 0.793 mV and 0.771 mV, respectively (Table 2).

**Figure 3:**
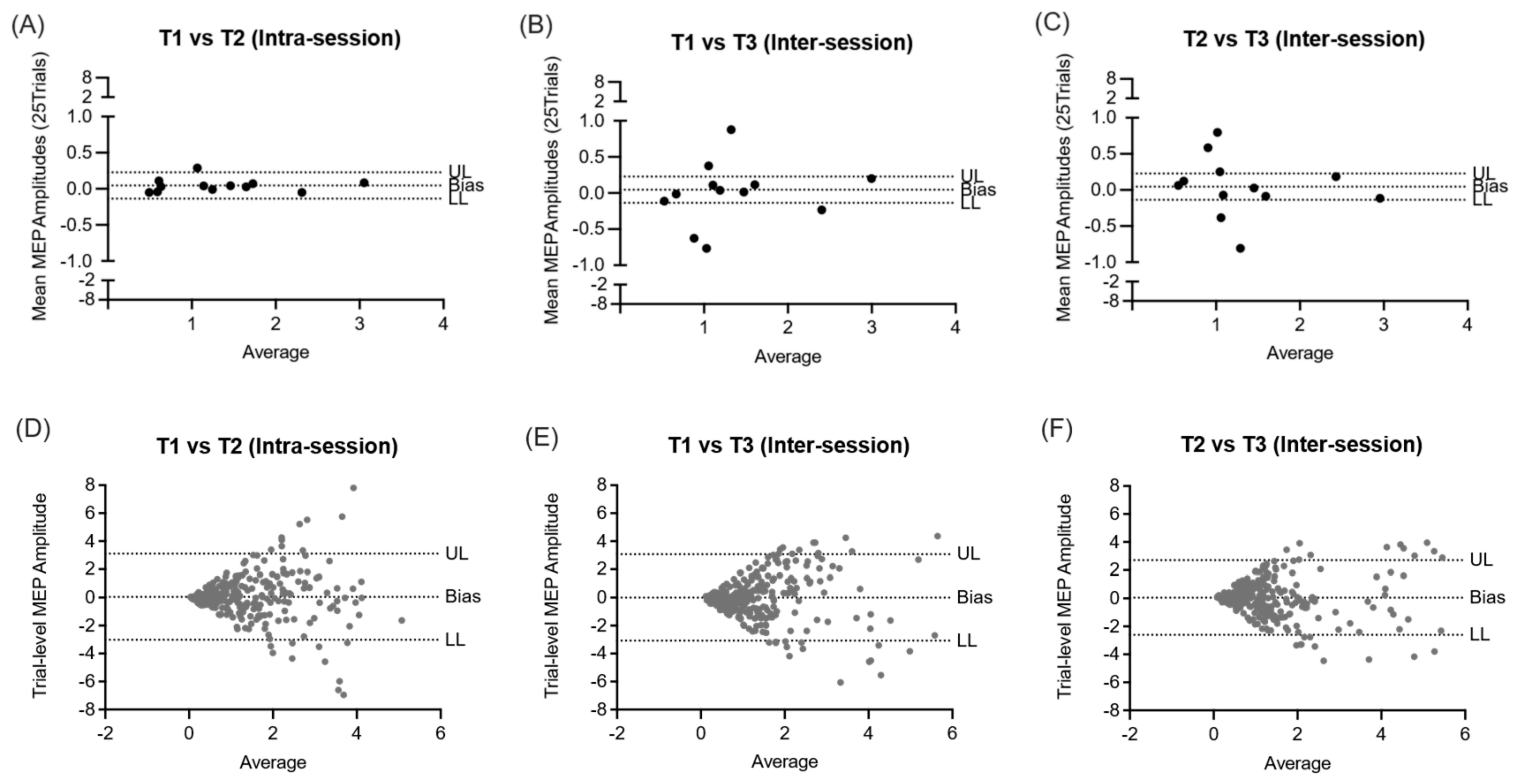
Bland–Altman plots of average and trial-level MEP amplitudes across repeated assessments. Panels (**A**–**C**) show Bland–Altman analyses based on the 25-trial mean MEP amplitude for each participant, comparing T1 vs. T2, T1 vs. T3, and T2 vs. T3, respectively. Panels (**D**–**F**) show the corresponding trial-level Bland–Altman analyses using individual MEP responses. For each plot, the x-axis represents the average of the paired MEP amplitudes, and the y-axis represents the difference between the paired measurements. The central horizontal line indicates the mean bias, while the upper and lower horizontal lines indicate the 95% limits of agreement. T1 vs. T2 represents intra-session agreement, whereas T1 vs. T3 and T2 vs. T3 represent inter-session agreement.

Bland–Altman analyses showed minimal mean bias across all comparisons (Figure 3). The intra-session comparison demonstrated the narrowest limits of agreement between T1 and T2 (bias = 0.045 mV; 95% LoA, −0.137 to 0.227 mV). Inter-session comparisons between T1 and T3 (bias = −0.003 mV; 95% LoA, −0.844 to 0.839 mV) and between T2 and T3 (bias = −0.048 mV; 95% LoA, −0.860 to 0.765 mV) showed wider limits of agreement. No proportional bias was detected in any comparison.

### 3.2. Trial-Level Reliability

Trial-level LMM analyses showed no significant differences in MEP amplitudes between timepoints (Table 1). Pairwise comparisons showed no significant differences for T1 vs. T2 (β = 0.045 mV, 95% CI: −0.159 to 0.250, t (885) = 0.520, *p* = 0.862), T1 vs. T3 (β = −0.003 mV, 95% CI: −0.207 to 0.202, t (885) = −0.029, *p* = 1.000), or T2 vs. T3 (β = −0.048 mV, 95% CI: −0.252 to 0.157, t (885) = −0.548, *p* = 0.848).

Trial-level analyses revealed substantial within-participant variability across repeated measurements. Reliability across the three timepoints was low, with an ICC of 0.304, CV of 78.982%, and MDC95 of 2.933 mV. Intra-session reliability between T1 and T2 was also low, with an ICC of 0.253, CV of 83.005%, and MDC95 of 3.060 mV. Inter-session reliability was similarly low for T1 vs. T3 (ICC = 0.224, CV = 81.826%, MDC95 = 3.070 mV), although reliability was slightly higher for T2 vs. T3 (ICC = 0.435, CV = 71.864%, MDC95 = 2.651 mV) (Table 2). These findings indicate that individual MEP trials were not stable standalone estimates of CSE, but they captured variability that was largely removed by averaging.

Bland–Altman analyses showed minimal mean bias across all comparisons (Figure 3), but wider limits of agreement than the average-level analyses. The intra-session comparison demonstrated the narrowest limits of agreement (LoA) between T1 and T2 (bias = 0.045 mV; 95% LoA, −3.018 to 3.108 mV). Inter-session comparisons between T1 and T3 (bias = −0.003 mV; 95% LoA, −3.076 to 3.071 mV) and between T2 and T3 (bias = −0.048 mV; 95% LoA, −2.702 to 2.607 mV) showed similarly wide limits of agreement. No proportional bias was detected in any comparison.

### 3.3. Exploratory Sequential Block Analyses

Exploratory sequential block analyses showed that reliability was lower and less consistent when the 25-trial recording was divided into early, middle, and late blocks. At the block-averaged level, no timepoint comparison reached statistical significance, indicating no consistent systematic change across repeated assessments within individual blocks (Table 1). However, reliability estimates were weaker than those obtained from the full 25-trial average. Overall block-level ICCs ranged from 0.514 to 0.668, intra-session ICCs ranged from 0.721 to 0.806, and inter-session ICCs were more variable, ranging from 0.181 to 0.759. CV and MDC95 values were also larger for block-level averages than for the full 25-trial average, suggesting that shorter sequential blocks provided less stable estimates of CSE (Table 2). At the trial level, block-specific reliability remained low overall, with ICCs ranging from 0.200 to 0.387 across the three timepoints and CVs ranging from 69.236% to 86.964%. Most block-specific trial-level timepoint comparisons were non-significant, although Block 3 showed a significant difference between T1 and T3 (β = −0.403 mV, 95% CI: −0.686 to −0.121, *p* = 0.016). Together, these exploratory findings support the trial-level results by indicating that full-block averaging provided the most reliable estimate while also being less sensitive to within-block temporal variability.

### 3.4. Bayesian Trial-Level Variance Decomposition

The Bayesian hierarchical model supported these findings and further showed why trial-level modelling was informative. The posterior mean differences between timepoints were small, and the 95% CrI crossed zero (T2 vs. T1: β = −0.046 mV, 95% CrI: −0.209 to 0.123, P [β > 0] = 0.299; T3 vs. T1: β = 0.005 mV, 95% CrI: −0.157 to 0.170, P [β > 0] = 0.517). The trial order showed a small positive posterior effect (β = 0.011 mV; 95% CrI: 0.001 to 0.020; P [β > 0] = 0.984). Variance decomposition indicated that residual/trial-level variability was larger than between-subject variability, with a posterior ICC of 0.336 (95% CrI: 0.186 to 0.550), consistent with the lower trial-level reliability estimates (Table 3). Thus, trial-level variability represented a major component of the data structure rather than a minor source of random error.

### 3.5. Influence of Participant Characteristics on MEP Amplitudes

Exploratory LMMs were conducted to examine whether participant characteristics were associated with trial-level MEP amplitudes while accounting for repeated observations within participants (Table 4). No statistically significant associations were observed for age, BMI, or gender. Age showed little association with MEP amplitude (β = −0.014 mV, 95% CI: −0.072 to 0.043, *p* = 0.585). BMI showed a negative trend (β = −0.102 mV, 95% CI: −0.226 to 0.023, *p* = 0.097), and gender also showed a non-significant trend (β = −0.902 mV, 95% CI: −1.937 to 0.133, *p* = 0.079), with female participants tending to have lower MEP amplitudes than male participants.

## 4. Discussion

This study addressed a methodological question that has become increasingly important as MEP analyses move beyond averaged responses: whether reliable estimation of CSE should rely on averaged MEP amplitudes, or whether trial-level data provide essential information about the structure and interpretation of the response. The main contrast was between averaged and trial-level analyses, with exploratory sequential block analyses used to examine temporal structure within the recording sequence. Although averaging 25 MEPs yielded reliable summary estimates, trial-level and sequential block analyses revealed substantial within-participant variability and evidence of temporal structure across the recording sequence. Thus, the present findings support a complementary interpretation: averaged MEP amplitude is useful for stable outcome measurement, whereas trial-level modelling is necessary for evaluating what the average may conceal.

The strong reliability of averaged MEP amplitude supports its continued use when the aim is to estimate stable CSE or detect intervention-related change. The excellent intra-session reliability observed between T1 and T2 suggests that, under standardized testing conditions, 25-trial averaged MEP amplitude can provide a precise estimate over short intervals. This finding is consistent with the rationale for collecting multiple MEP amplitudes per block, as averaging across trials reduces random trial-to-trial fluctuations and provides a more stable estimate of the underlying response tendency [25]. However, high reliability of the mean should not be interpreted as evidence that the underlying trial-level responses are homogeneous, normally distributed, or stationary across the recording sequence.

Inter-session reliability was lower than intra-session reliability but remained good, highlighting an important distinction between short-term measurement stability and longer-term reproducibility. While participants largely maintained their relative ranking in CSE across sessions, absolute agreement declined over the 48-h interval, as reflected by wider limits of agreement and larger MDC95 values. This pattern suggests that day-to-day variability may not substantially alter between-individual differences but can reduce sensitivity for detecting small within-individual changes over time [26]. From a clinical and experimental perspective, these findings indicate that longitudinal intervention studies should interpret small between-session changes cautiously, as observed differences may reflect normal biological and measurement variability rather than true neurophysiological adaptation [27]. Changes that exceed the MDC95 threshold are therefore more likely to represent meaningful physiological effects [12,28].

The trial-level findings provide an important complementary perspective. Although individual MEP amplitudes showed no systematic differences across timepoints, their reliability was substantially lower than the averaged MEP estimates. This distinction is important because the absence of mean bias does not necessarily imply that the underlying trial-level data are stable or well represented by a single average [29]. Low trial-level reliability indicates that individual MEPs should not be treated as standalone stable indicators of CSE. However, it does not imply that trial-level data lack value. Instead, trial-level analyses reveal whether the mean is supported by a stable distribution of responses or whether it is influenced by large fluctuations, extreme responses, non-stationarity, or changes in variability. This is particularly important for intervention studies, because an average-level pre-post change may appear statistically straightforward while the underlying trial-level data show heterogeneity that affects physiological interpretation.

This distinction also has important implications for statistical modelling. Conventional repeated-measures ANOVA often requires trial-level responses to be averaged before analysis, effectively collapsing within-block variability into a single summary estimate. This approach remains appropriate when the primary goal is to estimate mean CSE or detect robust intervention-related change [30]. In contrast, LMMs and Bayesian hierarchical models allow individual trials to be retained while appropriately accounting for their nesting within participants [31]. These models do not make single-trial MEPs inherently more reliable, but they allow researchers to model trial-level variability, trial order effects, block-dependent changes, and participant-specific response patterns directly. Therefore, the value of trial-level analysis lies not in replacing averaged MEP amplitude as a reliability metric, but in evaluating whether the averaged estimate is statistically and physiologically meaningful.

The exploratory sequential block analyses further support this interpretation. Dividing the recording into early, middle, and late blocks reduced reliability compared with the full 25-trial average, indicating that shorter portions of the recording were more vulnerable to trial-to-trial variability. At the same time, the block findings demonstrate why the average alone may be incomplete: reliability and response stability were not uniform across the recording sequence, and the significant Block 3 trial-level difference between T1 and T3 suggests that late-block responses may be more sensitive to temporal fluctuations in some contexts. This finding should be interpreted cautiously because the block analyses were exploratory and involved smaller numbers of observations per block. Nevertheless, the block-level results support the trial-level interpretation by showing that averaging can improve stability while simultaneously concealing within-block temporal features of the MEP response.

Exploratory analyses found no significant associations between MEP amplitude and participants’ characteristics at the trial level. These findings should be interpreted cautiously because the sample was not powered to examine demographic moderators. Nevertheless, they suggest that the primary reliability findings were unlikely to be strongly driven by the measured participant characteristics. Future studies with larger and more diverse samples should examine whether demographic, anatomical, hormonal, or behavioural factors influence both averaged and trial-level MEP reliability.

Practically, these findings suggest a two-level analytical workflow for MEP studies. Averaged MEP amplitude can be retained as the primary outcome when the research question concerns stable CSE estimation or change detection. However, trial-level inspection and modelling should be used to evaluate the response distribution, identify temporal patterns, and determine whether the average is an adequate representation of the underlying data. Exploratory block analyses can support this process by identifying whether trial-order structure is present within the recording sequence. When the research question concerns variability, state-dependent fluctuations, or individual response dynamics, LMMs or hierarchical models are better aligned with the nested structure of trial-level MEP data than analyses that require all responses to be averaged first.

### Limitations

The sample size was small and included only healthy, right-hand-dominant adults, limiting generalizability to older adults, clinical populations, or individuals with altered corticospinal excitability. The study focused on MEP amplitudes recorded from the FDI at 120% RMT, and reliability may differ across muscles, stimulation intensities, contraction states, and TMS protocols. Although testing was standardized and sessions were scheduled at the same time of day, factors such as sleep, physical activity, stress, hormonal status, and attention were not fully quantified. In addition, 25 MEPs were selected based on prior evidence, but the study did not directly compare different trial counts. The sequential block analyses were exploratory, used unequal block sizes because 25 trials could not be divided evenly into three equal segments, and should not be interpreted as a formal comparison of alternative trial-count protocols. Although the EMG recording site and TMS site were carefully replicated across two sessions, the possibility of experimenter-dependent variability cannot be fully excluded. Finally, although the present findings highlight the importance of trial-level structure, the study was not designed to provide a full distributional modelling framework for MEP amplitudes; future studies should examine skewness, extreme responses, and alternative distributional assumptions more directly. Individual trials across sessions do not represent repeated measurements of identical physiological events; therefore, low trial-level reliability should be interpreted as evidence of high response variability rather than failure of the TMS protocol.

## 5. Conclusions

The central issue is not whether averaged or trial-level MEP analysis is superior, but what each level of analysis is estimating. A 25-trial average provides a reliable estimate of participant-level CSE and remains appropriate for summary-based comparisons of sessions, groups, or interventions. Yet reliability of the mean does not establish reliability of the response process that produced it. Averaging stabilises the estimate by suppressing trial-to-trial fluctuation, but it also conceals dispersion, temporal drift, and block-dependent structure. Trial-level modelling is therefore not a substitute for averaging, nor are individual MEPs reliable standalone indicators of CSE. Rather, trial-level modelling tests the measurement validity of the average by revealing whether it reflects a stable response process or a compressed mixture of variable and temporally structured responses. Future studies should choose the data structure and model from the research question: averaged MEPs for estimating stable CSE, and hierarchical trial-level models for interrogating variability, temporal structure, and physiological interpretability.

## Figures and Tables

**Table 1:** LMM results of peak-to-peak MEP amplitudes.

Sessions	Comparison	β Estimate, mV	95% CI, mV	t (df)	*p* Value
Average-level LMM results					
Intra-	T1 vs. T2	0.045	−0.208 to 0.298	t (22) = 0.450	0.895
Inter-	T1 vs. T3	−0.003	−0.255 to 0.250	t (22) = −0.025	1.000
Inter-	T2 vs. T3	−0.048	−0.301 to 0.205	t (22) = −0.474	0.884
Block 1	T1 vs. T2	0.109	−0.245 to 0.464	t (24) = 0.636	0.531
Block 1	T1 vs. T3	0.193	−0.161 to 0.548	t (24) = 1.124	0.272
Block 1	T2 vs. T3	0.084	−0.271 to 0.439	t (24) = 0.489	0.63
Block 2	T1 vs. T2	0.175	−0.303 to 0.654	t (24) = 0.757	0.456
Block 2	T1 vs. T3	0.253	−0.225 to 0.731	t (24) = 1.091	0.286
Block 2	T2 vs. T3	0.077	−0.401 to 0.556	t (24) = 0.334	0.741
Block 3	T1 vs. T2	−0.127	−0.669 to 0.415	t (24) = −0.485	0.632
Block 3	T1 vs. T3	−0.403	−0.945 to 0.138	t (24) = −1.536	0.138
Block 3	T2 vs. T3	−0.276	−0.818 to 0.266	t (24) = −1.051	0.304
Trial-level LMM results					
Intra-	T1 vs. T2	0.045	−0.159 to 0.250	t (885) = 0.520	0.862
Inter-	T1 vs. T3	−0.003	−0.207 to 0.202	t (885) = −0.029	1.000
Inter-	T2 vs. T3	−0.048	−0.252 to 0.157	t (885) = −0.548	0.848
Block 1	T1 vs. T2	0.109	−0.147 to 0.366	t (24) = 0.636	0.402
Block 1	T1 vs. T3	0.193	−0.063 to 0.450	t (24) = 1.124	0.139
Block 1	T2 vs. T3	0.084	−0.172 to 0.340	t (276) = 0.839	0.520
Block 2	T1 vs. T2	0.175	−0.139 to 0.490	t (276) = 1.484	0.273
Block 2	T1 vs. T3	0.253	−0.061 to 0.567	t (276) = 0.645	0.114
Block 2	T2 vs. T3	0.077	−0.237 to 0.392	t (276) = 1.099	0.628
Block 3	T1 vs. T2	−0.127	−0.410 to 0.155	t (312) = 1.583	0.376
Block 3	T1 vs. T3	−0.403	−0.686 to −0.121	t (312) = 0.485	0.016 *
Block 3	T2 vs. T3	−0.276	−0.558 to 0.006	t (312) = −0.887	0.111

Average-level analyses were performed using participant-level mean MEP amplitudes at each timepoint, whereas trial-level analyses retained all individual MEP trials. β estimates represent mean differences between timepoints. Positive β values indicate higher MEP amplitudes at the later time point in each comparison. *p* values were adjusted for multiple comparisons using Tukey correction. Block 1, Block 2, and Block 3 represent the early, middle, and late phases of each MEP recording, respectively. Each recording consisted of 25 consecutive MEP trials, which were divided into three sequential blocks: Block 1 = trials 1–8, Block 2 = trials 9–16, and Block 3 = trials 17–25. MEP = motor evoked potential; LMM = linear mixed-effects model; * = *p* < 0.05.

**Table 2:** Reliability of MEP amplitudes across time points.

Sessions	Timepoint	ICC	CV	MDC95
Average-level results				
Overall	T1 vs. T2 vs. T3	0.899	17.735	0.642
Intra-	T1 vs. T2	0.992	4.935	0.191
Inter-	T1 vs. T3	0.844	22.393	0.793
Inter-	T2 vs. T3	0.852	22.004	0.771
Block 1	T1 vs. T2 vs. T3	0.514	35.522	1.183
Block 1	T1 vs. T2	0.806	23.677	0.821
Block 1	T1 vs. T3	0.243	47.838	1.622
Block 1	T2 vs. T3	0.453	30.891	0.985
Block 2	T1 vs. T2 vs. T3	0.523	45.058	1.594
Block 2	T1 vs. T2	0.790	31.407	1.172
Block 2	T1 vs. T3	0.459	45.648	1.670
Block 2	T2 vs. T3	0.181	56.784	1.904
Block 3	T1 vs. T2 vs. T3	0.668	46.274	1.835
Block 3	T1 vs. T2	0.721	38.546	1.375
Block 3	T1 vs. T3	0.518	54.758	2.250
Block 3	T2 vs. T3	0.759	42.771	1.799
Trial-level results				
Overall	T1 vs. T2 vs. T3	0.304	78.982	2.933
Intra-	T1 vs. T2	0.253	83.005	3.060
Inter-	T1 vs. T3	0.224	81.826	3.070
Inter-	T2 vs. T3	0.435	71.864	2.651
Block 1	T1 vs. T2 vs. T3	0.287	69.236	2.375
Block 1	T1 vs. T2	0.342	69.759	2.478
Block 1	T1 vs. T3	0.282	70.939	2.451
Block 1	T2 vs. T3	0.208	66.571	2.187
Block 2	T1 vs. T2 vs. T3	0.200	86.964	3.172
Block 2	T1 vs. T2	0.240	90.265	3.427
Block 2	T1 vs. T3	0.148	82.848	3.071
Block 2	T2 vs. T3	0.198	87.250	3.000
Block 3	T1 vs. T2 vs. T3	0.387	77.276	3.136
Block 3	T1 vs. T2	0.218	85.731	3.176
Block 3	T1 vs. T3	0.258	84.004	3.482
Block 3	T2 vs. T3	0.615	62.290	2.689

Reliability was evaluated at both the average level (average mean of 25 MEPs per timepoint) and the trial level (individual MEP responses retained). ICC values indicate relative reliability, CV reflects within-subject variability, and MDC95 represents the minimum change required to exceed measurement error at the 95% confidence level. Each recording consisted of 25 consecutive MEP trials, which were divided into three sequential blocks: Block 1 = trials 1–8, Block 2 = trials 9–16, and Block 3 = trials 17–25. MEP = motor evoked potential; ICC = intraclass correlation coefficient; CV = coefficient of variation; MDC95 = minimal detectable change at the 95% confidence level.

**Table 3:** Bayesian hierarchical regression of trial-level MEP amplitudes.

Parameter	Posterior Mean	95% CrI	P (β > 0)
Fixed-effect results			
Intercept	1.3618	0.8782 to 1.8519	1.0000
T2 vs. T1	−0.046	−0.2093 to 0.1227	0.2992
T3 vs. T1	0.0048	−0.1567 to 0.1704	0.5172
Trial order	0.0107	0.0009 to 0.0202	0.9840
Variance decomposition			
Between-subject variance	0.6185	0.2619 to 1.3906	—
Residual/trial-level variance	1.1410	1.0385 to 1.2496	—
Total variance	1.7594	1.3787 to 2.5362	—
Posterior ICC	0.3358	0.1864 to 0.5503	—

Posterior estimates are presented for fixed effects, variance components, and the posterior intraclass correlation coefficient (ICC). T1 was used as the reference time point for comparisons with T2 and T3. The fixed effect of trial order represents the linear change in MEP amplitude across the 25-trial recording sequence. Variance decomposition separates variability attributable to between-subject differences and residual trial-level variability. Values are reported as posterior means with 95% credible intervals (CrI). P (β > 0) indicates the posterior probability that the parameter estimate is greater than zero.

**Table 4:** Associations between participant characteristics and MEP amplitudes.

Predictor	Estimate	SE	95% CI	t	*p* Value
Intercept	4.721	1.675	0.858 to 8.584	2.818	0.023
Age	−0.014	0.025	−0.072 to 0.043	−0.569	0.585
BMI	−0.102	0.054	−0.226 to 0.023	−1.881	0.097
Gender	−0.902	0.449	−1.937 to 0.133	−2.009	0.079

Linear mixed-effects models were used, with participant included as a random effect to account for repeated MEP observations within individuals. Age and BMI were entered as continuous subject-level predictors, and gender was included as a categorical subject-level predictor. Estimates are unstandardised regression coefficients. For age and BMI, estimates represent the expected change in MEP amplitude in mV per one-unit increase in the predictor. For gender, the estimate represents the mean difference in MEP amplitude relative to the reference group. These analyses were exploratory and should be interpreted cautiously given the small sample size. MEP = motor evoked potential; BMI = body mass index.

## Data Availability

The data presented in this study are available from the corresponding author upon reasonable request. The data are not publicly available due to privacy concerns.

## Supplementary Materials

The following supporting information can be downloaded at: https://www.mdpi.com/article/10.3390/brainsci16090911/s1, Table S1: Resting motor threshold across assessment timepoints and sessions.
