# Supplementary material for: Intra- and Inter-Session Reliability of Average- and Trial-Level Motor Evoked Potential Amplitudes
Source: Brain Sci. 2026 Aug 27;16(9):911. doi: 10.3390/brainsci16090911 (PMC13604774; doi:10.3390/brainsci16090911)
Supplement: Supplementary file 1 [file brainsci-16-00911-s001.zip › brainsci-4478012-supplementary.pdf]

Table S1. Resting motor threshold across assessment timepoints and sessions

Subject	Session 1 (T1 and T2)	Session 2 (T3)	Session 2 - Session 1
1	33	36	3
2	43	38	-5
3	39	44	5
4	36	35	-1
5	31	35	4
6	29	29	0
7	35	37	2
8	45	46	1
9	42	46	4
10	36	41	5
11	30	30	0
12	39	37	-2
Mean ± SD %	36.50 ± 5.23	37.83 ± 5.57	1.33 ± 3.08
Primary test	t (11) = 1.50, p = 0.162		
95% CI for mean difference	-0.63 to 3.29		
Wilcoxon sensitivity test	W = 14, p = 0.189		

Session 1 corresponds to T1/T2 because both assessments were performed within the same session and shared the same resting motor threshold (RMT). Session 2 corresponds to T3. The primary comparison was Session 2 minus Session 1 using a paired t-test; Wilcoxon signed-rank test was included as a sensitivity analysis.
